# Effect of Burn Sites (Upper and Lower Body Parts) and Gender on Extensive Burns’ Mortality

**Published:** 2015-03

**Authors:** Ali Akbar Mohammadi, Mohammad Reza Pakyari, Seyed Morteza Seyed Jafari, Ahmad Reza Tavakkolian, Hamid Reza Tolide-ie, Zahra Moradi, Masumeh Kherad

**Affiliations:** 1 Shiraz Burn Research Center, Division of Plastic and Reconstructive Surgery, Department of Surgery, Ghotbedin Burn Hospital, Shiraz University of Medical Sciences, Shiraz, Iran;; 2 Shiraz Burn Research Center, Ghotbedin Burn Hospital, Shiraz University of Medical Sciences, Shiraz, Iran;; 3 Faculty of Public Health, Gonabad University of Medical Sciences, Gonabad, Iran

**Keywords:** Body parts, Burns, Female, Male, Outcome

## Abstract

Our recent literature survey indicated a lack of clinical assessment of the influence of gender and site of burn injury on the outcome of patients with extensive burns. This report examines the effect of burn sites and gender on extensive burns’ mortality.

Data was gathered from 283 patients with burns larger than 65% of the total body surface area (TBSA) above the belt line or below the belt line; and without underlying diseases and inhalation burn injury. Patients were classified according to gender, site of injury (upper and lower body parts) and hospital stay period. Mortality rates of each category were then compared with each other. The hospital stay period in the female group was significantly higher compared with the male group (P<0.001) and the mortality rate among the female patients was higher compared with the male patients (P=0.004). Although the mortality rate in lower body part of the male group was significantly higher in comparison with the upper body part burn (P=0.001), there was no difference in mortality rate of upper versus lower body part in the female group. The mortality rate was generally higher among the female patients. Additionally, higher mortality rate was observed among male patients with lower body part burn compared with injuries of male patients with upper body part burn.

## Introduction

Burn traumas are among the most devastating condition worldwide. In the developing countries, burns constitute a major health problem due to high incidence of severe complications and limitation of financial resources.^1^^,^^2^ Despite advancements in burn care during the past decades, treatment of patients with extensive burns remains a major challenge.^3^ Wide variation in age, injury type, depth and site of burn are observed among burn victims.^4^^,^^5^ Clinical surveys found lower mortality after non thermal accidents among females compared with males.^6^

Different complications, morbidity, and mortality rate are the key motives for studying the difference between burns of the upper and lower body parts.^7^ The results of this study may lead to a different approach in burn injuries of the upper and lower body parts.^8^

To date, few clinical studies have focused on outcome following burn injury with respect to the age, gender and burned body parts. In the absence of reliable literature survey, several years of practical experience in a referral burn center have shown significant differences between the upper and lower extremity burns. We investigate some of these differences and their possible influences on the upper and lower body parts burns. The age, sex, outcome, prognosis, and complications of the burn patients and mortality related to the site of burns were evaluated in this study. 

## Materials and Methods

A retrospective survey was designed to study mainly hospital stay period and mortality rate of patients who were admitted during November 2005 to December 2006. The formula for comparison of proportions was used to determine the sample size, in which the mortality rate in the upper extremity group of a pilot sample considered as P=15%, d=10% (as effect size), α=0.05 and β=0.80. Finally, the sample size of 250 was determined.

$$n=\frac{{\left(Z_{1-\frac{\alpha }{2}}\sqrt{2\bar{P}\left(1-\bar{P}\right)}+Z_{1-\beta }\sqrt{P_{1}\left(1-P_{1}\right)+P_{2}\left(1-P_{2}\right)}\right)}^{2}}{d^{2}}$$

Among the 465 patients with both upper and lower body part burns admitted to Ghotbedin Burn Center, 283 patients fulfilled the inclusion criteria. Patients were divided into two groups, namely “upper group” (larger than 65% TBSA above the belt line) and “lower group” (larger than 65% of TBSA below the belt line). TBSA was calculated according to the Berkow diagram. A burn physician assisted with the survey and registered the desired data of each group daily. A comparison between these groups was done with respect to variables such as mortality, hospital stay period, sex, age group, and mean TBSA.

The exclusion criteria in this study were comorbidities such as diabetes mellitus, cardiovascular diseases, hypertension, and having inhalation burn injury.

Initially, the mortality rate, hospital stay period, and TBSA were studied in male/female group and in upper/lower group separately. Then the male upper/lower group and female upper/lower group were compared. Statistical analysis was done by utilizing SPSS software version 15.0 in addition to Mann-Whitney test and chi-square statistical tests. The statistical significance level of P value was set at 0.05. 

## Results

Among the 283 patients, 163 (57.6 %) were male and 120 (42.4%) were female. The mean age was 25.8±6.8 with no significant difference in the male and female groups (P=0.57). The mean burned TBSA was 31.4±8.3 and there was no significant difference between men and women (P=0.46). The hospital stay in the female group was more than male (P<0.001) (table 1). Considering all previously mentioned variables, the only significant factors related to mortality were sex and TBSA. The mortality rate among female patients was significantly higher than the male ones (25% vs. 15.3%, [OR=1.84 (95% CI: 1.02-3.31)], P=0.04). Higher TBSA was also significantly associated with more mortality rate (P<0.001).

**Table 1:** Comparison of continuous variables between men and women burn patients

**Variables**	**Men (n=163)**		**Women (n=120)**		P value*
	**Mean±SD**	**Median (min-max)**	**Mean±SD**	**Median (min-max)**	
Age (year)	25.9±6.3	25 (15-42)	25.7±7.3	25 (15-45)	0.57
Hospital stay (day)	17.4±14.6	13 (1-86)	24.9±18	19.5 (1-80)	<0.001
Burned TBSA (%)	31.1±8.2	30 (18-50)	31.8±8.5	30.75 (16-48.5)	0.46

*Mann-Whitney test were used

*Upper versus Lower Body Part Burn Results*

The patients were divided into upper and lower body parts burn groups. No statistical significance was found except for the days of hospital stay in the upper and lower groups which was significantly higher in patients with lower extremity burn [Med=22 (range: 1-86) vs. Med=14 (range: 1-80), P=0.013]. Comparison between the upper and lower body parts in female and male revealed that only in the male group the hospital stay period was significantly different (table 2).

**Table 2:** Comparison of continuous variables between upper and lower extremity burn patients

** Group** **Sex **	**Variables **	**Upper**		**Lower**		P value*
		**Mean±SD**	**Median (min-max)**	**Mean±SD**	**Median (min-max)**	
Male	Age (years)	26.37±6.48	25 (15-40)	24.82±6.02	24 (15-42)	0.202
	Hospital Stay (days)	14.35±11.06	12 (1-74)	26.92±19.61	24 (1-86)	<0.001
	Burned TBSA (%)	30.31±7.94	28 (18-50)	33.62±8.64	34 (20-49)	0.033
Female	Age (years)	26.02±7.24	25 (15-45)	25.00±7.79	24.5 (15-43)	0.393
	Hospital Stay (days)	25.35±17.33	21 (1-80)	23.84±20.07	18 (3-80)	0.378
	Burned TBSA (%)	32.62±8.55	31.5 (20-48.5)	29.84±8.20	28 (16-45)	0.113

*Mann-Whitney test were used

*Female versus Male Group Results*

The mortality rate and the site of burn were compared in the male and female groups separately. The male upper group showed to have the least mortality in comparison with the other three groups (13%). 

## Discussion

It is commonly accepted that there are regional variations between different parts of the body skin and the reaction of different sites to burn wounds are dissimilar. For instance, patients with burn wounds of the lower parts of the body develop more and more edema and deep vein thrombosis (DVT).^9^ The lower extremity burns tend to have more complications and long-term disabilities. Edema, which is very common in the leg and foot burns, delays the process of wound healing and postpones grafting time.^10^ The risk of developing deep vein thrombosis and pulmonary embolism in patients with lower extremity burns can be a major cause of death in such patients.^7^

This might explain the results of our study regarding prolonged hospital stay period in patients with lower extremity burns (P=0.013). 

While our study showed a less mortality rate in upper body part burns of the male group (13% vs. 22.5%), the mortality rate of upper body part burns in the female group was slightly higher in comparison with the lower body part burns (25.3% vs. 24.2%). This might be due to different features in the skin of the lower part of a female’s body, which is more susceptible to complications and mortality. Further studies should be carried out to evaluate and confirm such postulation.

Multiple studies have shown several differences in the skin features of each gender, and susceptibility of each gender to skin cancers and skin inflammation.^11^ Previous studies have shown that gender would influence the thickness of the different layers of skin. For instance, the subcutaneous adipose tissue and the epidermis are thicker (more than 10-fold) in women and conversely the dermis layer is thicker in men.^12^

The subcutaneous adipose tissue has less vascular supply in comparison with the other layers of the skin. Neovascularization (originating from vessels of fascia and forming a vascular network on the surface of subcutaneous adipose tissue) is a marker for proper grafting time. Having thicker adipose tissue in females is directly associated with taking longer time for grafting. This can explain a longer hospital stay period and subsequent complications such as infections, sepsis and mortality in the female group in our study. 

In animal studies, female mice were observed to have a higher morbidity than males, following burn, in both survival and immune function.^13^ Recent laboratory studies have shown that immune responses differ between male and female human genders. It is believed that sex hormones regulate immunity that may lead to differences in immune cell activation, infiltration, and cytokine formation during and after injury between the genders.^13^

On the other hand, burn causes a rise in estrogen levels in female mice, where levels of estrogen in burned male mice reach low levels.^14^ McGwin et al. stated that up to 60 years of age, mortality rates among females were over twice that of males; however, no difference was noted among those of 60 years of age or older. Causes and timing of death were similar for both males and females. Compared to males, women younger than 60 years of age with burn injuries seem to be more susceptible to death.^6^ O’Keefe et al. have identified an increased mortality risk in women of 30 to 59 years of age.^15^

Longer hospital stay period that was observed in the female group can cause several complications such as malnutrition, infection, and sepsis. These could be considered as another cause of higher mortality rates in the female group of our study.

The skin has enzymes that transform sex hormones such as dehydroepiandrosterone into their more potent forms (dihydrotestosterone). This changes the collagen content of the skin differently in male and female.^16^ Actually, collagen is the main support for the skin resistance and there is a direct and definite relationship between dermal thickness and skin collagen contents.^16^ This means more dermal thickness and a better blood supply in males, which could be another cause of shorter wound healing process in the male group of this study.

## Conclusion

Female gender is a risk factor for developing higher mortality rate. Complications and mortality rate are significantly higher in the lower body compared with the upper body in male patients. 
